# Baseline Estimates of Colorectal Cancer Screening Among Adults Aged 45 to 75 Years, Behavioral Risk Factor Surveillance System, 2022

**DOI:** 10.5888/pcd22.250175

**Published:** 2025-08-07

**Authors:** Sallyann Coleman King, Jessica King, Cheryll C. Thomas, Lisa C. Richardson

**Affiliations:** 1 Division of Cancer Prevention and Control, National Center for Chronic Disease Prevention and Health Promotion, Centers for Disease Control and Prevention, Atlanta, Georgia; 2 US Public Health Service, Washington, DC

## Abstract

Colorectal cancer (CRC) screening allows for early detection and prevention through removal of polyps. In 2021, the US Preventive Services Task Force updated recommendations to screen adults aged 45 to 75 years. We analyzed 2022 Behavioral Risk Factor Surveillance System data to establish baseline prevalence estimates for those eligible for screening aged 45 to 75, including those aged 45 to 49 years who are newly eligible. Only 61.4% of adults aged 45 to 75 were up to date with CRC screening, below the Healthy People 2030 target (72.8%). Public health and clinical systems can prioritize identifying and screening adults not up to date on screening to reduce CRC risk.

**Summary**

**What is already known on this topic?**

Colorectal cancer (CRC) screening allows for early detection and prevention of CRC. The starting age for CRC screening was lowered to 45 years in 2021.

**What is added by this report?**

We used the new screening recommendation and 2022 Behavioral Risk Factor Surveillance System data to present baseline estimates of newly eligible adults aged 45 to 49 who are up to date on CRC screening; approximately 2 in 3 such adults have never been screened for CRC.

**What are the implications for public health practice?**

Recommendations from health care professionals can increase CRC screening uptake for newly eligible adults.

## Objective

Colorectal cancer (CRC) is the fourth most common cause of cancer among men and women in the US (1). While screening provides the opportunity for early detection and prevention through the removal of polyps, many eligible people remain unscreened (2). Current recommendations support screening people aged 45 to 75 years to reduce the risk of CRC (3). Those who receive screening have been shown to have a lower risk of the disease (4,5). Understanding the characteristics of those who are not up to date with CRC screening may be important for creating new interventions and refining existing interventions that support screening uptake. We conducted this analysis by using the updated questions in the 2022 Behavioral Risk Factor Surveillance System (BRFSS) to establish baseline prevalence estimates for those eligible for screening aged 45 to 75 (including those aged 45‒49 years who are newly eligible) based on changes in the US Preventive Services Task Force (USPSTF) recommendations in 2021 (3). The following test types were measured in our analysis: high-sensitivity guaiac fecal occult blood testing (gFOBT) annually, fecal immunohistochemical test (FIT) annually, stool DNA-FIT every 1 to 3 years, colonoscopy every 10 years, computed tomography (CT) colonography every 5 years, flexible sigmoidoscopy every 5 years, and flexible sigmoidoscopy with FIT every 10 years plus a FIT annually. We also assessed progress in achieving the Healthy People 2030 CRC screening target of having 72.8% of all age-eligible people screened for CRC (6).

## Methods

The BRFSS is an annual, state-based, random-digit–dialed telephone survey of the civilian, noninstitutionalized adult population aged 18 years or older (7). BRFSS collects information on demographic characteristics, health risk behaviors, preventive health practices, and health care access in the US. CRC screening questions are part of the rotating core of questions administered by all health departments during even years. In 2021, the questions for CRC screening were modified to align with the questions used in the National Health Interview Survey, including modifications in wording, number of questions, and inclusion of questions on CT colonography and Cologuard (Exact Sciences Corporation). We analyzed data from the 2022 BRFSS to examine screening prevalence among adults currently recommended for screening (adults aged 45‒75 years) and assessed baseline prevalence for those aged 45 to 49 years who are newly recommended for screening in the 2021 USPSTF recommendation (3). We assessed selected variables on demographic characteristics and health care access, tabulated by 3-level variables on screening status (up to date on screening by any method; screened but not up to date; and never screened). People were considered to be up to date with CRC screening if they followed the USPSTF recommendation for CRC screening with any test type (3). Respondents who declined to answer or who answered “don’t know/not sure” were excluded from analysis. Demographic variables analyzed were age (45–49, 50–64, 65–75 years), sex (male, female), race and ethnicity (Hispanic, non-Hispanic American Indian or Alaska Native, non-Hispanic Asian or Native Hawaiian Islander, non-Hispanic Black, non-Hispanic White, non-Hispanic “Other” race or multiracial), educational attainment (did not graduate from high school, graduated from high school, attended college or technical school, and graduated from college or technical school), annual household income (<$15,000, $15,000 to <$35,000, $35,000 to <$50,000, $50,000 to <$75,000, and ≥$75,000), and metropolitan or nonmetropolitan residence at the county level.

The BRFSS design weight handles nonresponse bias (7). We used SAS-callable SUDAAN version 9.4 (RTI International) to account for the complex sampling design. Data were weighted to the age, sex, and racial and ethnic distribution of each state’s adult population by using intercensal estimates. Results were age-standardized to the 2000 US standard million population for groups aged 45 to 49, 50 to 64, and 65 to 74 years.

## Results

The overall response rate for the 2022 BRFSS was 45.1 %. Six in 10 (61.4%; 95% CI, 60.9%–61.8%) adults aged 45 to 75 years were up to date with CRC screening, accounting for almost 69.4 million people (Table). Of the adults aged 45 to 75 years, more had never been screened (32.3%) than screened but not up to date (6.3%). Of the 3 age groups, adults aged 65 to 75 years were the most likely to be up to date with screening (81.5%). Almost 30% (29.8%; 95% CI, 28.6%–31.0%) of those aged 45 to 49 years were up to date with screening. About 63% of women reported being up to date with CRC screening (62.8%; 95% CI, 62.1%–63.4%). Up-to-date screening for men was 60.0% (95% CI, 59.3%–60.6%). By race and ethnicity, 65.0% of non-Hispanic Black and 63.5% of non-Hispanic White adults reported up-to-date screening, compared with 52.4% of Hispanic adults. Screening prevalence was higher among adults who had higher educational attainment (67.2% [95% CI, 66.6%–67.9%] among those who graduated from college or technical school), had higher annual household income (67.2% [95% CI, 66.5%–67.8%] among those with an income of ≥$75,000), lived in a metropolitan area (61.9%; 95% CI, 61.4%–62.4%), reported having a health insurance plan (63.5%; 95% CI, 63.0%–63.9%), reported having a personal doctor (64.6%; 95% CI, 64.1%–65.1%), and had a routine checkup within the past year (66.4%; 95% CI, 65.8%–66.9%). Nearly one-third of women (30.6%; 95% CI, 30.0%–31.3%) and 34.1% (95% CI, 33.5%–34.7%) of men had never been screened for CRC. Although adults aged 45 to 49 years represent a small proportion of those eligible for screening in the US, almost two-thirds (65.7%; 95% CI, 64.5%–67.0%) had never been screened.

**Table:** Colorectal Cancer Screening, by Demographic Characteristics, Among Survey Respondents Aged 45 to 75 Years, Age-Standardized to US 2000 Standard Million Population, Behavioral Risk Factor Surveillance System, 2022

Characteristic	Screened and up to date^a^			Screened but not up to date			Never screened		
	No.^b^	Weighted no.^c^	% (95% CI)	No.^b^	Weighted no.^c^	% (95% CI)	No.^b^	Weighted no.^c^	% (95% CI)
**Total**	152,617	69,383,246	61.4 (60.9–61.8)	15,276	6,912,665	6.3 (6.1–6.5)	49,300	28,326,502	32.3 (31.9–32.8)
**Age, y**									
45–49	7,430	4,494,252	29.8 (28.6–31.0)	1,131	665,883	4.4 (3.9–5.0)	16,639	9,905,489	65.7 (64.5–67.0)
50–64	71,184	37,146,966	66.9 (66.3–67.6)	7,101	3,646,075	6.6 (6.3–6.9)	24,013	14,715,832	26.5 (25.9–27.1)
65–75	74,003	27,742,028	81.5 (80.8–82.1)	7,044	2,600,707	7.6 (7.3–8.0)	8,648	3,705,181	10.9 (10.4–11.4)
**Sex**									
Male	70,265	32,855,903	60.0 (59.3–60.6)	6,603	3,163,611	6.0 (5.6–6.3)	24,452	14,706,371	34.1 (33.5–34.7)
Female	82,352	36,527, 343	62.8 (62.1–63.4)	8,673	3,749,054	6.6 (6.3–6.9)	24,848	13,620,131	30.6 (30.0–31.3)
**Race and ethnicity**									
Hispanic	7,071	6,894,150	52.4 (50.6–54.1)	806	777,527	5.9 (5.1–6.8)	5,051	5,473,168	41.7 (40.0–43.4)
Non-Hispanic American Indian or Alaska Native	2,038	728,319	53.7 (49.3–58.0)	230	63,665	4.8 (3.5–6.7)	1,204	496,493	41.5 (37.3–45.8)
Non-Hispanic Asian or Native Hawaiian Islander	2,692	2,862,985	53.4 (50.2–56.5)	256	272,015	5.3 (4.0–6.9)	1,692	2,081,866	41.3 (38.3–44.4)
Non-Hispanic Black	11,923	8,413,031	65.0 (63.5–66.4)	859	620,920	5.0 (4.4–5.6)	3,996	3,257,386	30.1 (28.7–31.5)
Non-Hispanic White	122,850	47,149,588	63.5 (63.0–63.9)	12,413	4,802,221	6.8 (6.5–7.0)	34,808	15,421,667	29.8 (29.3–30.2)
Non-Hispanic “Other” race or multiracial	2,444	1,673,798	60.8 (57.5–64.0)	293	211,987	7.9 (5.9–10.5)	1,068	752,818	31.3 (28.5–34.3)
**Education level**									
Did not graduate from high school	5,899	5,696,915	46.6 (44.8–48.5)	740	749,102	6.0 (5.2–7.0)	4,466	4,959,768	47.4 (45.6–49.2)
Graduated from high school	32,430	16,494,296	58.2 (57.2–59.1)	3,589	1,710,288	6.2 (5.8–6.6)	13,102	7,598,646	35.7 (34.7–36.6)
Attended college or technical school	42,791	22,276,097	62.8 (62.0–63.7)	4,462	2,197,668	6.5 (6.0–6.9)	13,300	8,018,756	30.7 (29.9–31.5)
Graduated from college or technical school	71,198	24,718,061	67.2 (66.6–67.9)	6,459	2,245,607	6.4 (6.0–6.7)	18,273	7,654,521	26.4 (25.8–27.0)
**Annual household income, $**									
<15,000	6,622	3,274,572	51.8 (49.9–53.7)	979	405,526	6.7 (5.9–7.6)	3,486	2,116,888	41.5 (39.6–43.5)
15,000 to <35,000	24,210	11,019,227	53.9 (52.7–55.0)	2,930	1,320,788	6.6 (6.0–7.2)	9,549	5,790,075	39.5 (38.4–40.7)
35,000 to <50,000	15,838	6,568,178	58.2 (56.6–59.8)	1,765	710,105	6.5 (5.9–7.2)	4,898	2,669,320	35.3 (33.6–36.9)
50,000 to <75,000	22,805	9,327,911	63.2 (62.0–64.4)	2,257	907,721	6.2 (5.7–6.8)	6,227	3,137,356	30.6 (29.4–31.7)
≥75,000	58,806	27,546,973	67.2 (66.5–67.8)	4,794	2,347,583	5.8 (5.5–6.2)	17,639	9,889,429	27.0 (26.4–27.7)
**Metropolitan or nonmetropolitan**									
Metro	109,262	58,426,583	61.9 (61.4–62.4)	10,417	5,660,463	6.2 (5.9–6.5)	34,121	23,608,333	31.9 (31.4–32.4)
Nonmetro	43,318	10,954,718	58.7 (57.8–59.6)	4,853	1,251,808	6.8 (6.4–7.3)	15,161	4,716,487	34.5 (33.6–35.4)
**Have health plan**									
Yes	147,200	66,323,527	63.5 (63.0–63.9)	14,311	6,401,816	6.3 (6.1–6.6)	42,803	23,763,799	30.2 (29.8–30.7)
No	2,064	1,371,230	28.7 (26.0–31.6)	602	328,858	7.1 (4.8–10.4)	5,005	3,591,154	64.2 (61.3–66.9)
**Have personal doctor**									
Yes	145,820	65,799,662	64.6 (64.1–65.1)	13,690	6,106,705	6.2 (5.9–6.4)	39,080	22,140,978	29.2 (28.8–29.7)
No	6,048	3,170,164	33.3 (31.8–34.8)	1,467	755,831	7.9 (7.0–8.9)	9,741	5,853,127	58.8 (57.3–60.4)
**Last routine checkup**									
Never	153	81,394	18.0 (13.0–24.3)	47	27,842	7.1 (4.2–12.0)	568	342,469	74.9 (68.0–80.7)
Within past year	136,999	62,236,363	66.4 (65.8–66.9)	11,750	5,245,255	5.8 (5.5–6.0)	32,664	18,965,208	27.9 (27.4–28.4)
2 to <5 Years ago	13,542	6,203,889	46.8 (45.5–48.1)	2,576	1,157,080	8.8 (8.1–9.5)	10,032	5,814,278	44.4 (43.1–45.7)
≥5 Years ago	1,223	563,899	16.0 (14.3–17.8)	752	393,514	11.2 (9.4–13.3)	5,241	2,733,851	72.8 (70.5–75.1)

a Up to date with screening includes those screened by any of the screening tests recommended by US Preventive Services Task Force within the suggested screening interval (3): 1) high-sensitivity guaiac fecal occult blood testing annually, 2) fecal immunohistochemical test (FIT) annually, 3) stool DNA-FIT every 1 to 3 years, 4) colonoscopy every 10 years, 5) computed tomography colonography every 5 years, 6) flexible sigmoidoscopy every 5 years, or 7) flexible sigmoidoscopy with FIT every 10 years, plus FIT every year.

b Number who participated in the survey.

c Number who participated in the survey extrapolated to the US population.

## Discussion

A previous report showed that the up-to-date CRC screening prevalence among adults 50 to 75 years was approximately 72% (2). While we cannot make direct comparisons because our data comprised adults starting at age 45 years, our data showed that 61.4% of adults aged 45 to 75 were up to date on CRC screening, indicating that concerted and continued efforts can help reach the Healthy People 2030 target of 72.8%. However, less than one-third of adults aged 45 to 49 years were up to date, and an estimated 28 million adults aged 45 to 75 years were never screened. Continued improvement will be needed to meet the Healthy People 2030 objective of 72.8% (6) for adults aged 45 to 75 years being up to date with CRC screening (6).

Clinical recommendations have consistently been shown to increase cancer screening uptake (8). These recommendations could help with the almost one-third of age-eligible adults and the two-thirds of adults aged 45 to 49 who have never been screened for CRC. Because completing even a single screening with colonoscopy or sigmoidoscopy can provide some benefit in reducing CRC risk (5,9), clinicians can discuss all appropriate screening options with newly eligible patients so that they are prepared to follow through now and at regular intervals in the future (10). The National Health Interview Survey showed that most adults who were not up to date with screening reported that they did not receive a recommendation from their clinician at their most recent annual visit (8).

Our findings are subject to several limitations. BRFSS data are self-reported, which may lead to reporting bias (over and under reporting). Participants are recruited through a random-digit–dialing system, which excludes institutionalized people and some members of the military. These groups may have different levels of CRC screening uptake due to different levels of accessing care or different levels of trust in the health care system. Finally, CRC screening includes many options and various screening intervals, which can be complicated and difficult to recall accurately.

The new baseline estimate in 2022 of only 61.4% of those aged 45 to 75 years reporting being up to date with CRC screening creates a challenge in reaching 2030 Healthy People goals. Twenty-eight million American adults aged 45 to 75 years reported never being screened for CRC; of those, 35.0% were aged 45 to 49 years. Clinicians, public health practitioners, community health organizations, and others can work together to reduce barriers and help to ensure that each patient visit with a clinician includes time to address CRC screening. This concerted effort can help to ensure that those who are eligible receive screening services that may affect their long-term health.
